# ERC/Mesothelin Is Associated with the Formation of Microvilli on the Mesothelium and Has Limited Functional Relevance Under Physiological Conditions

**DOI:** 10.3390/ijms26094330

**Published:** 2025-05-02

**Authors:** Liang Yue, Kazunori Kajino, Toshiyuki Kobayashi, Yoshinobu Sugitani, Masami Sugihara, Soichiro Kakuta, Norihiro Harada, Hitoshi Sasano, Masataka Kojima, Masaaki Abe, Rong Lu, Naomi Otsuji, Akira Orimo, Okio Hino

**Affiliations:** 1 Department of Molecular Pathology, Juntendo University Graduate School of Medicine, 2-1-1 Hongo, Bunkyo-ku, Tokyo 113-8421, Japan; l-yue@juntendo.ac.jp (L.Y.); koba396@juntendo.ac.jp (T.K.); y-sugitani@juntendo.ac.jp (Y.S.); masa@juntendo.ac.jp (M.A.); kadowaki@juntendo.ac.jp (N.O.); aorimo@juntendo.ac.jp (A.O.); ohino@juntendo.ac.jp (O.H.); 2 Department of Human Pathology, Juntendo University Graduate School of Medicine, 2-1-1 Hongo, Bunkyo-ku, Tokyo 113-8421, Japan; lu.ok@juntendo.ac.jp; 3 Department of Pathology, Juntendo University Tokyo Koto Geriatric Medical Center, 3-3-20 Shinsuna, Koto-ku, Tokyo 136-0075, Japan; 4 Department of Clinical Laboratory Medicine, Juntendo University Graduate School of Medicine, 2-1-1 Hongo, Bunkyo-ku, Tokyo 113-8421, Japan; msugiha@juntendo.ac.jp; 5 Department of Clinical Laboratory, Juntendo University Tokyo Koto Geriatric Medical Center, 3-3-20 Shinsuna, Koto-ku, Tokyo 136-0075, Japan; 6 Laboratory of Morphology and Image Analysis, Biomedical Research Core Facilities, Juntendo University Graduate School of Medicine, 2-1-1 Hongo, Bunkyo-ku, Tokyo 113-8421, Japan; skakuta@juntendo.ac.jp; 7 Department of Respiratory Medicine, Juntendo University Graduate School of Medicine, 2-1-1 Hongo, Bunkyo-ku, Tokyo 113-8421, Japan; nor@juntendo.ac.jp (N.H.); h-sasano@juntendo.ac.jp (H.S.); 8 Department of Otorhinolaryngology, Juntendo University Graduate School of Medicine, 2-1-1 Hongo, Bunkyo-ku, Tokyo 113-8421, Japan; mkojima@juntendo.ac.jp

**Keywords:** electron microscopy, ERC/mesothelin, *Erc*-knockout mouse, microvilli

## Abstract

In adults, expressed in renal cancer (ERC)/mesothelin is exclusively expressed in the mesothelial cells lining the pleural, pericardial, and peritoneal cavities, yet its function under physiological conditions is unknown. To explore this, we studied ERC expression in wild-type (WT) mice at different developmental stages by immunohistochemistry and analyzed the ultrastructure of the mesothelium in WT and *Erc*-knockout (KO) mice via electron microscopy. Additionally, cardiopulmonary function in adult WT and *Erc*-KO mice was assessed using echocardiography and the forced oscillation technique (FOT). During embryonic development in WT mice, ERC expression was detected in the epicardium as early as embryonic day (E)12.5 but was absent in the pleura until E18.5. The timing of expression appeared to coincide with the active maturation of these organs, which implied a potential role in cardiopulmonary development. Electron microscopy revealed that microvilli on the mesothelium of *Erc*-KO mice were immature compared to those of WT mice. Based on these findings, we hypothesized that ERC might contribute to cardiopulmonary function; however, echocardiography and FOT did not reveal any functional differences between WT and *Erc*-KO mice. This suggests that ERC has limited functional relevance under physiological conditions.

## 1. Introduction

Expressed in renal cancer (ERC) was first identified in a renal cell carcinoma of an Eker rat [1,2] and is the homolog of human mesothelin (MSLN) [3] or megakaryocyte potentiating factor [4]. In normal tissues, ERC/mesothelin is specifically expressed in mesothelial cells lining the pleural, pericardial, and peritoneal cavities. The mesothelium originates from the mesodermal part of the gastrula, and the pattern of ERC expression during mesothelial development remains unelucidated. In malignancies, ERC is abundantly expressed in epithelioid mesothelioma, ovarian cancer, pancreatic cancer, and other cancers [5,6,7,8,9]. The overexpression of ERC in cultured cells enhances cellular adhesive or invasive activities [10,11,12]. However, its function in the normal mesothelium is not yet known. Bera and Pastan reported that *Erc*-knockout (KO) mice develop and reproduce normally without apparent abnormalities based on macroscopic and optical microscopic observation [13]. Later, Hilliard et al. found, by electron microscopic (EM) observation, that the microvilli on the peritoneal mesothelium of *Erc*-KO mice were shorter and more truncated compared to those of wild-type (WT) mice [14].

In this study, we examined ERC expression in WT mice at various developmental stages and analyzed the ultrastructure of the pleura and epicardium in *Erc*-KO mice using EM. We revealed that in the embryonic (E) stage of WT mouse development, ERC expression was detected in the epicardium as early as E12.5 but was absent in the pleura until E18.5. This timing coincided with the stages of cardiac and pulmonary maturation. ERC may be associated with cardiopulmonary function in mice by facilitating the stability of the pericardial and pleural cavities. Furthermore, our EM analysis revealed that microvilli on the pleural and pericardial mesothelium in *Erc*-KO mice were shorter and less dense compared to those in WT mice. Given these results, we hypothesized that the cardiopulmonary function of *Erc*-KO mice might be impaired due to the immature formation of microvilli. To test this hypothesis, we assessed cardiac function using echocardiography and pulmonary function via the forced oscillation technique (FOT) with a flexiVent™ system [15]. Nevertheless, no significant differences in cardiac or pulmonary function were observed between WT and *Erc*-KO mice. Therefore, although ERC is associated with microvillus formation on the mesothelium, its functional relevance to normal cardiopulmonary physiology appears to be limited.

## 2. Results

### 2.1. Expression of ERC in Adult Mice

**Figure 1:**
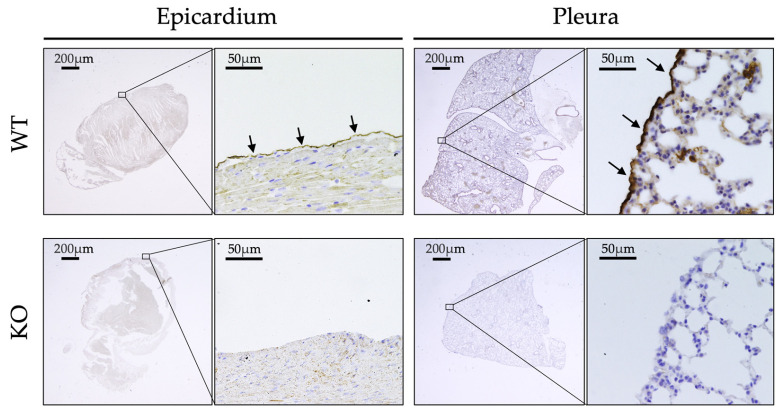
Expression of Expressed in renal cancer (ERC) in adult mice. ERC was found in the epicardium and pleura of wild-type (WT) but not of *Erc*-knockout (KO) mice. Arrows indicate the expressed ERC in the mesothelium of the epicardium and pleura.

Figure 1 shows the expression of ERC in 20-week-old mice as examined by immunohistochemistry (IHC). ERC was expressed in the epicardium and pleura of WT but not *Erc*-KO mice. Negative IHC staining in *Erc*-KO mice confirmed the specificity of the method. ERC was also expressed in the pericardium, peritoneum, and umbilical cord of WT mice. These results indicated that ERC was specifically detected in the mesothelium.

### 2.2. Expression of ERC During Developmental Stages of WT Mice

**Figure 2:**
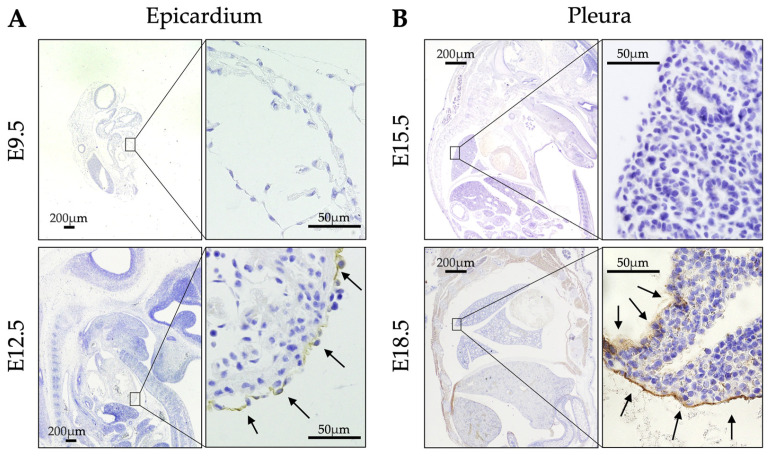
Expression of ERC in the developmental stages of WT mice. (**A**) In the epicardium, ERC was not expressed at embryonic day (E)9.5 but was expressed at E12.5. (**B**) In the pleura, it was not expressed at E15.5 but was expressed at E18.5. Arrows indicate the expressed ERC/mesothelin in the mesothelium of the epicardium and pleura.

In embryonic stages, ERC expression was not detected in the epicardium at E9.5 but was by E12.5 (Figure 2A). In the pleura, ERC expression was negative at E15.5 and positive at E18.5 (Figure 2B). Table 1 outlines the changes in ERC expression in fetuses and in post-delivery day (P)0 and P3 mice. Thus, ERC expression in the epicardium preceded that in the pleura by six days in WT mice.

### 2.3. Ultrastructural Differences in the Mesothelium Between WT and Erc-KO Mice

**Figure 3:**
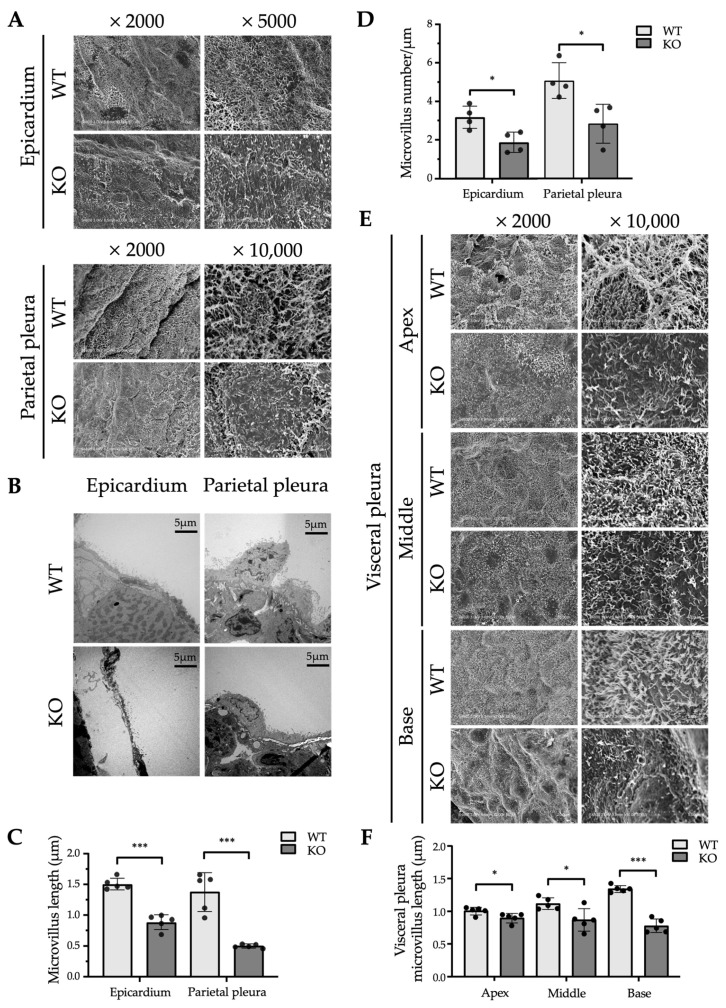
Ultrastructural difference in the mesothelium between WT and *Erc*-KO mice. (**A**,**B**) Scanning electron microscopy (SEM) (**A**) and transmission electron microscopy (TEM) of (**B**) images of the mesothelium in the epicardium and parietal pleura of WT and *Erc*-KO mice are shown with magnification (SEM: epicardium, ×2000, ×5000; parietal pleura ×2000, ×10,000. TEM: ×1000). (**C**) Comparison of the microvillus length in the mesothelium of the epicardium and parietal pleura between WT (*n* = 5) and *Erc*-KO mice (*n* = 5), based on measurements from SEM images (shown in (**A**)), with twenty microvilli measured per mouse. (**D**) Comparison of the microvillus density (number of microvilli per micrometer of mesothelial surface) in the epicardium and parietal pleura between WT (*n* = 4) and *Erc*-KO (*n* = 4) mice, based on measurements from TEM images (shown in (**B**)). (**E**) SEM images of the mesothelium in the apical (apex), middle, and basal (base) portions of the visceral pleura of WT and *Erc*-KO mice are shown with magnification (×2000, ×10,000). (**F**) Comparison of microvillus length in the apical (apex), middle, and basal (base) regions of the visceral pleura between WT (*n* = 5) and *Erc*-KO (*n* = 5) mice, based on measurements from SEM images (shown in (**E**)), with twenty microvilli measured per mouse. Each dot represents the mean value of twenty microvillus lengths in one mouse. Values are shown as mean ± standard deviation (SD). * *p* < 0.05, *** *p* < 0.001.

Figure 3 illustrates the ultrastructure of the microvillus-covered surface of mesothelial cells in WT and *Erc*-KO mice. Both scanning electron microscopy (SEM; Figure 3A) and transmission electron microscopy (TEM; Figure 3B) revealed that many microvilli were significantly shorter and less dense in *Erc*-KO compared to those in WT mice (Figure 3C,D). Because of the previously known morphological variation in microvilli in various regions of the pleura [16,17], we compared the length of microvilli in the apex (cranial portion), middle, and basal parts (caudal portion) of the visceral pleura in WT and *Erc*-KO mice. In all portions of the lung, the length of the microvilli was shorter in *Erc*-KO than in WT mice (Figure 3E,F). In WT, the microvilli were longer on the basal than on the apical side (base vs. middle, *p* < 0.01; middle vs. apex, *p* < 0.05); however, this tendency was not observed in *Erc*-KO mice (base vs. middle, ns; middle vs. apex, ns; Figure 3F). In *Erc*-KO mice, microvilli were shorter both in the epicardium and pleura than in WT mice; the regional difference in microvilli of the pleura found in WT mice was not observed.

### 2.4. Ultrastructural Differences in the Mesothelium Between Human Mesothelioma Cell Lines

**Figure 4:**
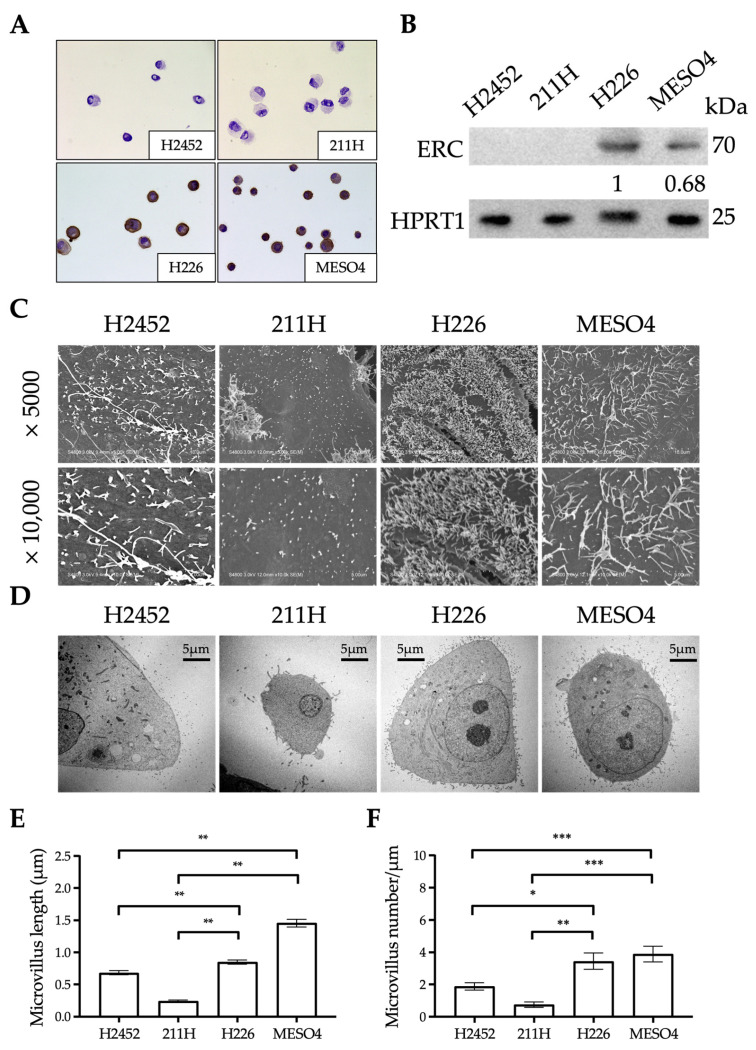
Expression of ERC and ultrastructure of microvilli in four human mesothelioma cell lines. (**A**) Immunocytochemistry results for ERC/mesothelin are shown. Nuclei were counterstained with hematoxylin. (**B**) Results of Western blotting to detect ERC (upper panel) and hypoxanthine-guanine phosphoribosyltransferase 1 (HPRT1) as an internal control (bottom panel) in four human mesothelioma cell lines. The numbers below the ERC blot represent the relative protein expression levels of ERC, as determined by densitometric analysis using ImageJ (version 1.54b). (**C**,**D**) SEM (**C**) and TEM (**D**) images of four human mesothelioma cell lines are shown with magnification (SEM; ×5000, ×10,000. TEM; ×1000). (**E**) The lengths of 100 microvilli, measured from SEM images (shown in (**C**)), were compared between four mesothelioma cell lines. (**F**) Microvillus densities (microvillus number/µm of mesothelial surface), measured from TEM images (shown in (**D**)), were compared between four mesothelioma cell lines. Values are shown as means ± standard error. * *p* < 0.05, ** *p* < 0.01, *** *p* < 0.001.

We examined the relationship between ERC expression and the ultrastructure of microvilli in four human mesothelioma cell lines [18]: NCI-H2452 (H2452), MSTO-211H (211H), NCI-H226 (H226), and ACC-MESO-4 (MESO4). Immunocytochemistry and Western blotting detected the endogenous expression of ERC in H226 and MESO4 but not in H2452 or 211H cell lines (Figure 4A,B). The two ERC-positive cell lines showed statistically longer and denser microvilli than the two negative cell lines (Figure 4C–F). ERC was therefore suggested to also be involved in the maturation of microvilli in cultured cells.

### 2.5. Cardiopulmonary Function of WT and Erc-KO Mice

**Figure 5:**
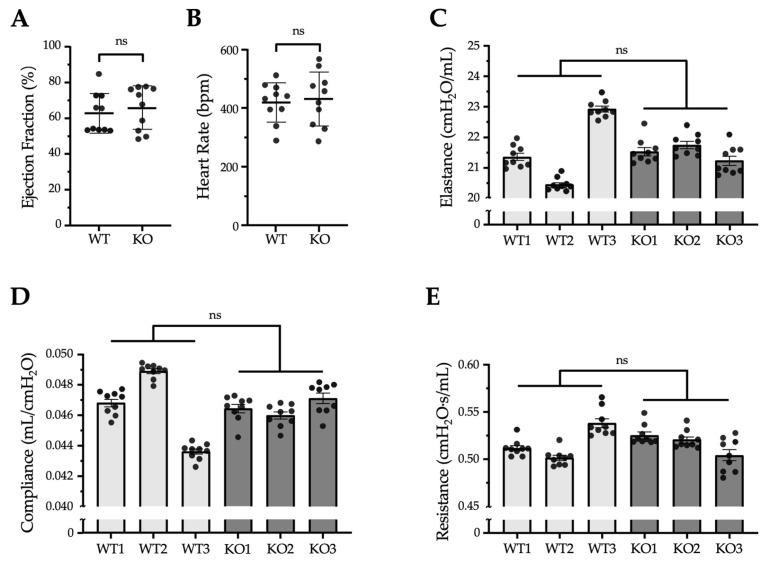
Cardiopulmonary functions of WT and *Erc*-KO mice. (**A**,**B**) Ejection fractions (**A**) and heart rates (**B**), analyzed by Vevo^®^ 2100 echocardiography (Fujifilm Visual Sonics Primetech Corporation, Toronto, ON, Canada), were compared between WT (*n* = 10) and *Erc*-KO (*n* = 10) mice. Each dot represents the measurement obtained from an individual mouse. Values are means ± SD. bpm, beats per minute. (**C**–**E**) Respiratory elastance (**C**), pulmonary compliance (**D**), and resistance (**E**) were analyzed in each mouse using a flexiVent™ system and compared between WT (*n* = 3) and *Erc*-KO (*n* = 3) mice. Measurements were repeated nine times per mouse. Each dot represents an individual measurement. Values are shown as mean ± standard error. Statistical comparisons between WT and *Erc*-KO groups were performed using the mean value per mouse (WT vs. KO mean ± SD: (**C**) 21.5824 ± 1.2516 vs. 21.5071 ± 0.2578 cmH_2_O/mL; (**D**) 0.0464 ± 0.0227 vs. 0.0465 ± 0.0006 mL/cmH_2_O; (**E**) 0.5171 ± 0.0190 vs. 0.5168 ± 0.0111 cmH_2_O·s/mL). ns: not significant.

We speculated that morphologically abnormal microvilli in the pleura or epicardium would have several disadvantageous effects on the cardiopulmonary function of *Erc*-KO mice. To evaluate this, we assessed such functions in both WT and *Erc*-KO mice. First, we examined the cardiac shape and function of WT (*n* = 10) and *Erc*-KO (*n* = 10) mice by echocardiography. By macroscopic observation and B (brightness)-mode echocardiography, we did not find any apparent malformation of the heart in *Erc*-KO mice. M (motion)-mode analysis showed that the diameters of the left ventricle in the systolic phase (WT 2.59 ± 0.55 vs. *Erc*-KO 2.49 ± 0.57 mm) and diastolic phase (WT 3.90 ± 0.45 vs. *Erc*-KO 3.88 ± 0.39 mm) did not differ significantly between the two groups of mice, which was reflected in the ejection fractions (Figure 5A). Measurements were taken under anesthesia, and changes in heart rate in response to the same concentration of isoflurane were also comparable between the two groups (Figure 5B). Next, we assessed the pulmonary size and function in WT and *Erc*-KO mice. Lung size was evaluated by measuring the height and width of the thorax, with no significant differences observed in these parameters between the two groups (height of lung: WT 1.95 ± 0.12 vs. *Erc*-KO 1.80 ± 0.17 cm, width of bilateral lungs: WT 1.85 ± 0.07 vs. *Erc*-KO 1.78 ± 0.18 cm). Pulmonary function was studied using a single-frequency force oscillation technique (FOT) with a flexiVent™ system (SCIREQ, Montreal, QC, Canada), which measures the pulmonary elastance, compliance, and respiratory resistance of the mouse lung. We found no significant differences in these three functional parameters between WT and *Erc*-KO mice (Figure 5C–E). Thus, our hypothesis that cardiopulmonary function is impaired in *Erc*-KO mice was not validated.

## 3. Discussion

The function of ERC in normal tissues is currently unknown. If a function exists, it would likely be exerted on organs associated with the mesothelium, where ERC is specifically expressed. For clues on the role of ERC, we studied the morphological details of tissues expressing ERC. We examined the expression of ERC during developmental stages of mice and the ultrastructure of the mesothelium in adult mice. We used *Erc*-KO mice, established previously [19], for three purposes in this study: First, organs of *Erc*-KO mice were used as negative controls in the IHC staining of ERC to confirm the specificity of the antibody (Figure 1). Second, the ultrastructure of mesothelial cells in the epicardium and parietal pleura was compared between WT and *Erc*-KO mice. Third, the cardiopulmonary function was compared between WT and *Erc*-KO mice to evaluate the physiological significance of ERC deficiency.

Figure 2 and the Table 1 reveal that the developmental stage in which ERC starts to be expressed differed between the pleura and epicardium. Expression was observed at E12.5 in the epicardium and at E18.5 in the pleura. During mouse development, the heart starts to beat between E8 and E9, when the shape of the heart is like a tube. This primitive form of the heart subsequently changes during maturation. Ventricular trabeculation, as well as valve and epicardium formation, occur at E11–12.5 [20,21]. We detected ERC in the epicardium at E12.5, and this timing almost coincided with that of epicardium formation. As for the lung movements of fetal mice, Niblock et al. reported that movements were reliably observed at E16.5, and that they increased significantly by E18.5 [22]. We detected ERC in the pleura at E18.5, and this timing almost coincided with the timing of increased lung movements. Since the timing of ERC expression in the epicardium and pleura seemed to coincide with maturation stages of the heart and lungs, respectively, we hypothesized that ERC is functionally associated with cardiopulmonary function in mice. In a previous study of the mesothelium and hedgehog signaling, Dixit et al. showed that ERC is expressed at E11.5, E12.5, and E14.5 in the epicardium and is not expressed on these embryonic days in the pleura of mice [23]. Their findings are similar to ours, although they did not mention the relationships between the timing of ERC expression and the maturation stages of these organs.

Figure 3A–D shows the morphological differences in microvilli in the epicardium and parietal pleura between WT and *Erc*-KO mice. In *Erc*-KO mice, many microvilli were shorter and of lower density compared to those of WT mice. Figure 4A–E reveals that the microvilli of cultured cells with negative expression of ERC were shorter and sparser than those in cells that showed endogenous expression. Hilliard et al. previously reported similar findings in the peritoneal mesothelium using mesothelin-KO mice established by Bera and Pastan [13]. They described how the microvilli on the peritoneal mesothelium of mesothelin-KO mice were truncated compared to those of WT mice [14]. Their study and ours showed that the shorter or truncated microvilli in *Erc*-KO mice were not organ specific but commonly seen in organs associated with the mesothelium, suggesting that ERC is one of the factors required for the maturation of microvilli in the mesothelium.

The mesothelial cells in the epicardium or pleura are exposed to shear stress from the pericardial or pleural fluid during heartbeats or respiratory movements, respectively [20,24]. It is proposed that microvilli, together with glycoproteins produced by the mesothelium, constitute a layer of serous exudate that creates a slippery cushion to protect the mesothelium from damage by friction [25]. We hypothesized that the function of ERC is to assist in cardiopulmonary function by maintaining longer and denser microvilli on the mesothelium.

Studies using the pleura of sheep and rabbits show that the morphology of mesothelial cells is not uniform in all parts of the pleura and that microvilli are longer in the caudal (basal) than the cranial (apical) portion of the visceral pleura [16,17]. It was suggested that the length of microvilli is possibly associated with pulmonary function because longer microvilli were observed in the base of the lung, which undergoes greater movement during respiration. We compared the lengths of microvilli in the apical (cranial portion), middle, and basal parts (caudal portion) of the visceral pleura of WT and *Erc*-KO mice. It was found that in any portion of the lung, microvilli were shorter in *Erc*-KO than in WT mice (Figure 3E,F). In WT mice, longer microvilli were observed at the base of the lung, which moves more widely than the apex. In contrast, this tendency was lost in *Erc*-KO mice, in which the lengths of microvilli were almost the same in the apex, middle, and base.

Meanwhile, Figure 4 reveals that ERC deficiency led to abnormalities in microvillus morphology. The four mesothelioma cell lines we analyzed showed different microvillus structures. Cells lacking endogenous ERC had visibly different microvilli compared to ERC-positive cells. We also noticed that ERC expression levels varied among the cell lines in both immunocytochemistry and Western blotting (Figure 4A,B). However, since only four cell lines were analyzed, the evidence remains preliminary. In future work, we plan to select suitable cell lines and establish inducible ERC expression models. This will allow us to study more precisely how ERC levels influence microvillus structure.

The findings shown in Figure 3 and Figure 4 suggest that *Erc*-KO mice may have several functional defects in the heart or lungs. We compared the shape and function of both organs between WT and *Erc*-KO adult mice. In the heart, B-mode echocardiography did not reveal any structural abnormalities in *Erc*-KO mice. M-mode did not show any differences in ventricular sizes, ejection fractions, and heart rates (Figure 5A,B) between the two groups. As for pulmonary function, Figure 5C–E shows a lack of difference in elastance, compliance, and resistance between WT and *Erc*-KO mice.

We were not able to validate our hypothesis that the cardiopulmonary function of *Erc*-KO mice was affected by the formation of immature microvilli in the pleura and epicardium. These results are compatible with the findings reported by Bera and Pastan, who observed the normal development and reproduction of *Erc*-KO mice [13], although they did not use EM or cardiopulmonary function tests. It seems that, under physiological conditions, ERC is dispensable or redundant in the maintenance of the normal life of mice, but we cannot conclude this yet. The methods we used in this study were limited, and if we are able to examine more fine functions such as the electrophysiological activities of the heart or lung, it is still possible that we will find some differences between WT and *Erc*-KO mice. While no significant cardiopulmonary abnormalities were observed up to 20 weeks of age, long-term effects of ERC deficiency cannot be ruled out. Longer-term studies are needed to clarify its physiological role. Pulmonary function tests were conducted using three female mice to minimize animal sacrifice based on the absence of significant sex differences in IHC, EM, and cardiac assessments. However, the exclusive use of female mice may represent a limitation of this study. Future studies should ensure a balanced representation of both sexes. Under pathological conditions, on the other hand, two research groups revealed the function of ERC by comparing the experimental outcomes observed in WT and *Erc*-KO mice. Zhang et al. reported that spontaneously developed renal carcinoma in *Tsc2*-KO mice is smaller in double-KO mice (*Tsc2*-KO x *Erc*-KO) than in single-KO mice (*Tsc2*-KO x WT) [19]. According to Hilliard et al., a mouse ovarian cancer cell line injected into the peritoneal space of *Erc*-KO mice showed decreased metastasis compared to that injected into WT mice [14]. Both reports support the idea that ERC accelerates the growth or metastasis of tumors in vivo and indicate that ERC behaves like an oncogenic factor in tumorigenesis.

In conclusion, we showed that microvilli in the pleura and pericardium were immature in *Erc*-KO mice. We hypothesized that cardiopulmonary function in *Erc*-KO mice would be impaired because of immature microvilli. Our hypothesis was not validated by a cardiopulmonary examination using echocardiography and FOT. These results imply that ERC may have limited functional relevance under physiological conditions. In injuries or inflammation, mesothelial cells are reported to secrete inflammatory cytokines and play roles in tissue repair [26,27]. Our future work is aimed to examine whether ERC works as a stress-responsive factor, such as those activated during inflammation, injuries, or situations associated with cardiopulmonary failures, to protect organs under these pathological conditions.

## 4. Materials and Methods

### 4.1. Animals and Tissue Preparation

We performed all in vivo studies according to the guidelines of the Laboratory Animal Experimentation Committee of Juntendo University School of Medicine (Approval Number: 2020158). All mice in this study were kept under specific pathogen-free conditions in a temperature- and humidity-controlled facility (23 ± 1 °C, 55 ± 5% humidity) with a 12 h light/dark cycle. Water and food were freely available. *Erc*-KO mice, which were previously established [19], were used for three main purposes in this study. First, tissues from *Erc*-KO mice were used as negative controls in ERC IHC staining to validate the specificity of the anti-ERC antibody (Figure 1). Second, electron microscopy was performed to assess and compare the ultrastructural features of mesothelial cells between WT and *Erc*-KO mice. Third, cardiopulmonary function was assessed in both WT and *Erc*-KO mice to investigate the physiological impact of ERC deficiency under normal conditions. Genotyping to confirm WT or *Erc*-KO mice was performed by PCR of genomic DNA using the following primers (sense and antisense): TACACAGTAGCCAGCATACC and ATTCCAGACCTGGCACTTCA for the wild-type allele and GCCCGGTTCTTTTTGTCAAG and ATACTTTCTCGGCAGGAGCA for the mutant allele. Adult 20-week-old WT and *Erc*-KO mice were euthanized by cervical dislocation, and the lungs were inflated before fixation as described previously [28]. In brief, a 22-gauge soft catheter (0.9 mm in diameter) was inserted into the trachea with a 2.5 mL syringe, and then 4% paraformaldehyde (PFA) was injected at a rate of flow no higher than ~200 µL/second until the lungs were fully inflated. After lung inflation, we held the catheter in place for a few more seconds to prevent the backflow of the fixative out of the trachea and then withdrew the catheter. The inflated lungs were removed and fixed in 4% PFA for IHC analysis. For EM, the lungs were removed without inflation and fixed in 2.5% glutaraldehyde (TAAB Laboratory Equipment, Aldermaston, UK). We also examined ERC expression in the fetuses of WT mice. After the pairing of 12-week-old WT C57/BL6 female and male mice, females were checked every morning for the presence of a vaginal plug. The day a plug was observed was designated E0.5. Pregnant mice were euthanized at E9.5, E12.5, E15.5, and E18.5 (*n* = 3 per stage), and fetuses were isolated and fixed in 4% PFA. The organs of mice were also harvested on post-delivery days P0 and P3 and at 20 weeks (*n* = 3 per each stage). For IHC, dissected organs of mice or whole-body fetuses were immediately fixed in 4% PFA at 4 °C for 6 h.

### 4.2. Cell Lines

The human mesothelioma cell lines NCI-H2452 (H2452), NCI-H226 (H226), and MSTO-211H (211H) were obtained from the American Type Culture Collection (Rockville, MD, USA). The cell line ACC-MESO-4 (MESO4), which was established at the Aichi Cancer Research Center Institute [18], was obtained from the RIKEN BioResource Center (RIKEN BRC; Tokyo, Japan). All cell lines were cultured in RPMI-1640 medium supplemented with 10% fetal calf serum at 37 °C in a 95% air/5% CO_2_ atmosphere.

### 4.3. Immunohistochemistry

Three-micrometer-thick tissue sections were prepared from PFA-fixed and paraffin-embedded specimens. After deparaffinization, tissue sections were treated with 3% hydrogen peroxide. For antigen retrieval, tissue sections were placed in Citrate Buffer Antigen Retriever pH 6.0, heated in a decloaking chamber (Biocare Medical, Irvine, CA, USA) at 95 °C for 20 min, cooled to 70 °C, removed from the chamber, and allowed to cool to about 40 °C. The sections were then blocked with 5% normal goat serum. After blocking, tissue sections were incubated with 0.5 μg/mL primary antibody (anti-mouse C-ERC/mesothelin (308), IBL, Fujioka, Japan) overnight at 4 °C. Then, sections were incubated at room temperature for 60 min with a horseradish peroxidase-labeled polymer anti-rabbit (Envision K4003; Dako, Glostrup, Denmark) secondary antibody. Finally, the slides were incubated in 3,3-diaminobenzidine for 2–3 min. In embryos at E12.5 and later stages, the surface mesothelial cells covering the heart and lungs were identified using a light microscope to locate the epicardium and pleura. ERC immunoreactivity was observed in the epicardium but not in the pleura until E18.5. At E9.5 and earlier, anatomical structures were immature, and the entire embryos showed negative ERC staining in our analysis. This negativity is related to the fact that the epicardium is not formed before E11-12.5 [20]. ERC positivity was assessed by comparison with negative controls. A tissue sample was considered ERC-positive if ERC staining was observed in the mesothelial regions. In all positive cases, the majority of mesothelial cells within the stained area exhibited clear ERC immunoreactivity.

### 4.4. Immunocytochemistry

Cells of the four cultured mesothelioma cell lines were smeared on glass slides and air dried for 30 min. The cell smears were fixed with acetone for 10 min at room temperature, followed by 1% PFA in phosphate-buffered saline (PBS) at 4 °C for 3 min and then treated with 3% hydrogen peroxide in PBS at room temperature for 10 min. Non-specific binding sites were blocked by incubation with 5% normal goat serum in PBS for 15 min at room temperature. Next, slides were incubated with 2 µg/mL mouse monoclonal anti-human ERC antibody, 22A31 (IBL, Gunma, Japan) [29], diluted in 1% bovine serum albumin and 0.05% NaN_3_ in PBS at 4 °C overnight. After washing with PBS, the cells were incubated with goat anti-mouse Ig conjugated with peroxidase-labeled dextran polymer (Envision K4001; Dako, Glostrup, Denmark) at room temperature for 60 min. Diaminobenzidine was used as the peroxidase substrate. The cells were counterstained with hematoxylin.

### 4.5. Western Blotting of Cellular Lysates

Cells were harvested 48 h after passage. The cellular lysate (50 µg) was adjusted in a solution containing 2% sodium dodecyl sulfate, 10% glycerol, 50mM Tris-HCl (pH 6.8), and 100mM dithiothreitol. After boiling for 2 min, the samples were electrophoresed on 10% Laemmli gels and transferred to polyvinylidene fluoride membranes (Immobilon-P, Merck Millipore, Carrigtwohill, Ireland). Proteins on the membranes were blocked in 1% skim milk in PBS with 0.1% Tween-20 (PBS-T) for 1 h at room temperature. The membranes were then incubated with 0.5 µg/mL mouse monoclonal anti-human ERC antibody (22A31), as described previously [29]. Goat anti-mouse Ig conjugated with Envision K4001 was used as the secondary antibody at a dilution of 1:100 in 1% skim milk with PBS-T at room temperature for 1 h. An Enhanced Chemiluminescence detection system (GE Healthcare, Buckinghamshire, UK) was employed to visualize the proteins on membranes. The signal was detected using a ChemiDoc MP imaging analyzer (Bio-Rad, Tokyo, Japan). The expression level of hypoxanthine-guanine phosphoribosyltransferase 1 (HPRT1) (ab109201, Abcam Limited, Cambridge, UK) was used as the internal control for Western blotting.

### 4.6. EM and Morphometry

Samples for SEM were processed as previously described [30]. In brief, tissues from WT and *Erc*-KO mice (*n* = 5 per group, three males and two females each), as well as mesothelioma cells, were fixed with 2.5% glutaraldehyde in 0.1 M phosphate buffer (pH 7.4), followed by post-fixation with 2% osmium tetroxide in the same buffer. After dehydration with a graded series of ethanol, specimens were transferred to t-butyl alcohol and freeze dried in an ES-2030 freeze dryer (Hitachi High Technologies, Tokyo, Japan). After mounting on aluminum stubs with carbon paste, the dried specimens were coated with osmium using an OPC80T osmium plasma coater (Filgen, Nagoya, Japan) and observed with an S-4800 high-resolution field emission SEM (Hitachi High Technologies). Microvillus length was measured using SEM images, as previously described [31]. Microvilli that were clearly visible and in focus were selected per sample from ×5000 magnification images. Each microvillus was traced along its full contour using the freehand line tool in ImageJ (version 1.54b) (National Institutes of Health, Bethesda, MD, USA), and the length was recorded with the Measure function. As microvilli are three-dimensional structures, their apparent length and number may be influenced by their orientation relative to the imaging plane, and we assumed that these influences occur to the same extent both in WT and KO mice. To minimize measurement bias, we performed multiple measurements and selected the most appropriate plane for analysis.

Samples for TEM were processed as previously described [30]. In brief, WT and *Erc*-KO mouse (*n* = 4 per group, two males and two females each) tissues and mesothelioma cells were fixed with 2.5% glutaraldehyde in 0.1 M phosphate buffer (pH 7.4), followed by post-fixation with 2% osmium tetroxide in the same buffer. Fixed specimens were dehydrated with a graded series of ethanol and embedded in epoxy resin (Oken Epok 812; Oken-shoji, Tokyo, Japan). Ultrathin sections were cut with a diamond knife, transferred to copper grids (150 mesh) that had been coated with Formvar membrane, stained with uranyl acetate and lead citrate, and observed with an HT7700 TEM (Hitachi High Technologies). For the assessment of microvillus density, TEM images at ×1000 magnification were used. The apical surface of mesothelial cells was traced with the freehand line tool in ImageJ (version 1.54b), and the surface length was calculated based on the image scale bar. Microvilli were counted by marking each one with the multi-point tool, and density was calculated as the number of microvilli per unit surface length.

### 4.7. Assessment of Cardiac Function in WT and Erc-KO Mice

We performed non-invasive transthoracic echocardiography (Vevo^®^ 2100; Fujifilm Visual Sonics Primetech Corporation, Toronto, ON, Canada) in sedated mice as previously described [32,33]. Ten 20-week-old mice (five males and five females) from each WT and *Erc*-KO group were anesthetized with 2% isoflurane. Then, mice were placed in a supine position on a temperature-controlled surgical table to maintain temperature at 37 °C with continual electrocardiogram monitoring via limb electrodes. We measured the ejection fraction percentage and heart rate from the left ventricular M-mode at the mid-papillary muscle level.

### 4.8. Assessment of Pulmonary Function in WT and Erc-KO Mice

For the evaluation of the size and function of lungs, 20-week-old WT and *Erc*-KO mice were used. As for the evaluation of lung size, thoracic height and width were measured in isoflurane-anesthetized WT (*n* = 5, three males and two females) and *Erc*-KO (*n* = 6, three males and three females) mice using a small animals computed tomography scanner (Latheta LCT-200; Aloka, Tokyo, Japan). Pulmonary function was measured by FOT using a flexiVent™ system (SCIREQ, Montreal, QC, Canada) as previously described [34]. The FOT is the gold standard for the examination of respiratory function in animals and is also used for human cases. The principle of FOT is described elsewhere [15]. Briefly, in a single-frequency FOT, an oscillatory airflow waveform is applied to a subject’s airway opening, and the subject’s responses, including pressure, flow, and volume signals, are analyzed by a single compartment model using linear regression. Three parameters, including respiratory system resistance, elastance, and compliance, are obtained. Respiratory resistance reflects airway resistance and respiratory tissue resistance. Elastance refers to the elastic stiffness of the lung contributed by the chest walls and airways. Compliance reflects the ease with which the respiratory system can be extended and is the reciprocal of elastance. Three 20-week-old female mice each from the WT and *Erc*-KO group were anesthetized with pentobarbital and xylazine and then intubated with metal 18-gauge catheters via tracheotomy. The catheter was then connected to a flexiVent™ system followed by measurements of respiratory resistance, pulmonary compliance, and elastance using flexiWare 7 software (SCIREQ). Each measurement was repeated nine times per mouse. The mice were mechanically ventilated with an average breathing frequency of 150 breaths/min.

### 4.9. Statistical Analysis

Statistical analysis was conducted using GraphPad Prism 8 (GraphPad Software Inc., San Diego, CA, USA). Both microvillus length and number in mesothelium tissue and cells and cardiopulmonary function measurements were compared using Student’s *t* test. Differences were considered statistically significant at *p* < 0.05.

## Figures and Tables

**Table 1:** Expressional changes in ERC in the epicardium and pleura during developmental stages of WT mice (fetal or neonatal).

	Stage	E9.5	E12.5	E13.5	E14.5	E15.5	E18.5	P0	P3
Site									
Epicardium		−	+	+	+	+	+	+	+
Pleura		−	−	−	−	−	+	+	+

E, embryonic day; ERC, expressed in renal cancer; P, post-delivery day; WT, wild-type; *n* = 3 per stage; ‘+’, ERC staining was positive at the corresponding site in all three mice (fetal or neonatal); ‘−’, ERC staining was negative at the corresponding site in all three mice (fetal or neonatal).

## Data Availability

The original contributions presented in this study are included in the article. Further inquiries can be directed to the corresponding author.

## Abbreviations

The following abbreviations are used in this manuscript:

B-mode	Brightness mode
bpm	Beats per minute
E	Embryonic day
EM	Electron microscopy
ERC/*Erc*	Expressed in renal cancer
FOT	Forced oscillation technique
HPRT1	Hypoxanthine-guanine phosphoribosyltransferase 1
IHC	Immunohistochemistry
KO	Knockout
M-mode	Motion mode
*MSLN*	Mesothelin
ns	Not significant
P	Post-delivery day
PBS-T	Phosphate-buffered saline with 0.1% Tween-20
PCR	Polymerase chain reaction
PFA	Paraformaldehyde
PBS	Phosphate-buffered saline
SD	Standard deviation
SEM	Scanning electron microscopy
TEM	Transmission electron microscopy
*Tsc2*	Tuberous sclerosis complex 2
WT	Wild-type
